# *N*-[(*R*)-(2-Chloro­phen­yl)(cyclo­pent­yl)meth­yl]-*N*-[(*R*)-(2-hydr­oxy-5-methyl­phen­yl)(phen­yl)meth­yl]acetamide

**DOI:** 10.1107/S1600536809044808

**Published:** 2009-10-31

**Authors:** Guang-You Zhang, Di-Juan Chen, Shu-Hong Wang, Ting Yang, Jian-Guo Chang

**Affiliations:** a School of Chemistry and Chemical Engineering, University of Jinan, Jinan 250022, People’s Republic of China; b Jincheng Pharmaceutical Co Ltd, Shandong Provience 255100, People’s Republic of China; c Department of Materials Science and Chemical Engineering, Taishan University, Taishan 271021, People’s Republic of China

## Abstract

In the title compound, C_28_H_30_ClNO_2_, the cyclo­pentane ring adopts an envelope conformation. In the crystal structure, mol­ecules are linked by inter­molecular O—H⋯O hydrogen bonds, forming chains running along the *a* axis.

## Related literature

For general background to amides, see: Calligaris *et al.* (1972 ▶); Ali *et al.* (2002 ▶); Cukurovali *et al.* (2002 ▶); Sriram *et al.* (2006 ▶); Kargar *et al.* (2009 ▶); Takenaka *et al.* (2002 ▶); Varlamov *et al.* (2003 ▶); Zhang *et al.* (2003 ▶). For the synthesis, see: Yang *et al.* (2005 ▶).
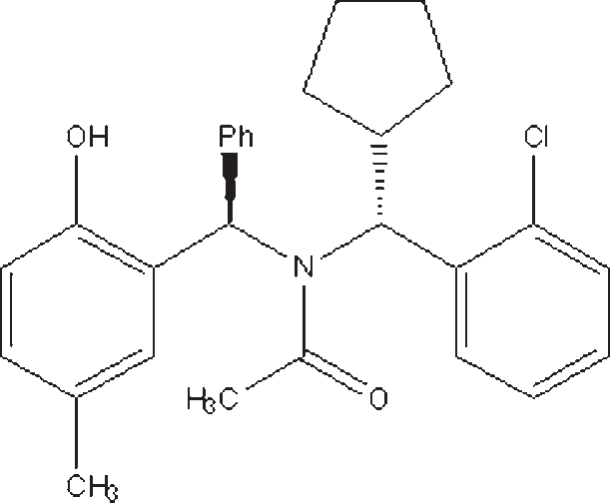


## Experimental

### 

#### Crystal data

C_28_H_30_ClNO_2_
*M* *_r_* = 447.98
Orthorhombic, 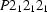

*a* = 8.8038 (8) Å
*b* = 11.3417 (10) Å
*c* = 25.485 (2) Å
*V* = 2544.7 (4) Å^3^
*Z* = 4
Mo *K*α radiation
μ = 0.17 mm^−1^
*T* = 298 K
0.24 × 0.16 × 0.12 mm

#### Data collection

Bruker SMART CCD area-detector diffractometer
Absorption correction: multi-scan (*SADABS*; Sheldrick, 1996 ▶) *T*_min_ = 0.960, *T*_max_ = 0.980
13334 measured reflections
4452 independent reflections
2845 reflections with *I* > 2σ(*I*)
*R*_int_ = 0.034

#### Refinement

*R*[*F* ^2^ > 2σ(*F* ^2^)] = 0.060
*wR*(*F* ^2^) = 0.186
*S* = 1.05
4452 reflections
290 parameters
H-atom parameters constrained
Δρ_max_ = 0.33 e Å^−3^
Δρ_min_ = −0.37 e Å^−3^
Absolute structure: Flack (1983 ▶), 1871 Friedel pairs
Flack parameter: 0.04 (16)

### 

Data collection: *SMART* (Bruker, 1999 ▶); cell refinement: *SAINT* (Bruker, 1999 ▶); data reduction: *SAINT*; program(s) used to solve structure: *SHELXTL* (Sheldrick, 2008 ▶); program(s) used to refine structure: *SHELXTL*; molecular graphics: *SHELXTL*; software used to prepare material for publication: *SHELXTL*.

## Supplementary Material

Crystal structure: contains datablocks I, global. DOI: 10.1107/S1600536809044808/xu2646sup1.cif

Structure factors: contains datablocks I. DOI: 10.1107/S1600536809044808/xu2646Isup2.hkl

Additional supplementary materials:  crystallographic information; 3D view; checkCIF report

## Figures and Tables

**Table 1:** Hydrogen-bond geometry (Å, °)

*D*—H⋯*A*	*D*—H	H⋯*A*	*D*⋯*A*	*D*—H⋯*A*
O1—H1⋯O2^i^	0.82	1.82	2.637 (3)	172

Symmetry code: (i) 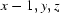.

**Fig. 1.**
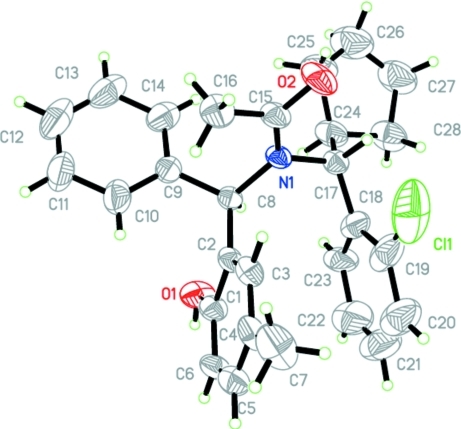
The structure of the title compound with 30% probability ellipsoids. H atoms are shown as spheres of arbitrary radii.

**Fig. 2.**
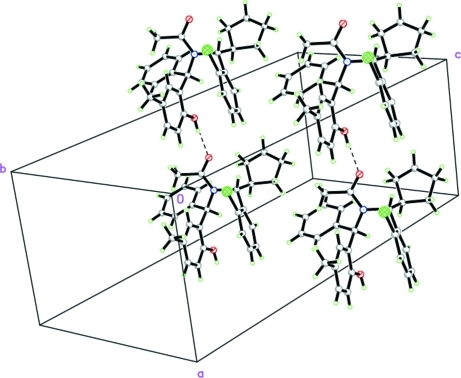
A packing diagram of (I), dashed lines represent hydrogen bonding.

## supplementary crystallographic information

### Comment

The Schiff base compounds have been widely used in organic and bioinorganic chemistry due to their significant biological activities (Ali *et al.*, 2002; Sriram *et al.*, 2006; Cukurovali *et al.*, 2002). These compounds have also been used as versatile ligands in coordination chemistry (Kargar *et al.*, 2009; Calligaris *et al.*, 1972). Therefore, the design and syntheis of Schiff bases with various functionalities has become an important field of research nowdays (Takenaka *et al.*, 2002; Varlamov *et al.*, 2003; Zhang *et al.*, 2003). As part of our continuing research of Schiff bases, we report the crystal structure of the title compound, which was acetylated from the corresponding aminophenol.

As shown in Fig. 1, the configuration at the new chiral center (C8) is *R*. The C9—C14 and C18—C23 aromatic rings are approximately vertical, the dihedral angle between their planes being 83.59 (17)°; the dihedral angle between the planes of the C1—C6 and C18—C23 aromatic rings is 17.45 (19)°, while that between the C1—C6 and C9—C14 planes is 78.89 (16)°. The intermolecular O1—H1···O2 hydrogen-bonding interactions are present in the crystal packing (Table 1 and Fig. 2).

### Experimental

The chiral aminophenol was prepared by a condensation reaction of (*R*)-1-(2-chlorophenyl)-1-cyclopentylmethanamine and (2-hydroxy-5-methylphenyl)(phenyl)methanone followed by reduction with NaBH_4_. The detailed procedure is similar to that reported by Yang *et al.* (2005). Then the aminophenol was reacted with acetic anhydride (5 equivalents) in a THF solution (10 ml) at room temperature for 12 h to obtain the title compound. After removal of the solvent, water (20 ml) was added to the residue, stirred and filtrated. Further purification of the filter cake was carried out by thin-layer silica-gel chromatography (chloroform) to give a colorless solid (yield 70.1%). Single crystals of the title compound suitable for X-ray analysis were obtained by slow evaporation of the n-hexane/ethyl acetate solution (3:2 *v*/*v*).

### Refinement

All H atoms were placed in geometrically idealized positions and constrained to ride on their parent atoms, with C–H = 0.93–0.98 Å and O–H = 0.82 Å, and refined in riding mode with *U*_iso_(H) = 1.5*U*_eq_(C,*O*) for methyl H atoms and hydroxy-H atoms, and 1.2*U*_eq_(C) for the others.

### Crystal data

**Table:**

C_28_H_30_ClNO_2_	*F*(000) = 952
*M**_r_* = 447.98	*D*_x_ = 1.169 Mg m^−^^3^
Orthorhombic, *P*2_1_2_1_2_1_	Mo *K*α radiation, λ = 0.71073 Å
Hall symbol: P 2ac 2ab	Cell parameters from 2267 reflections
*a* = 8.8038 (8) Å	θ = 2.5–19.1°
*b* = 11.3417 (10) Å	µ = 0.17 mm^−^^1^
*c* = 25.485 (2) Å	*T* = 298 K
*V* = 2544.7 (4) Å^3^	Block, colourless
*Z* = 4	0.24 × 0.16 × 0.12 mm

### Data collection

**Table:**

Bruker SMART CCD area-detector diffractometer	4452 independent reflections
Radiation source: fine-focus sealed tube	2845 reflections with *I* > 2σ(*I*)
graphite	*R*_int_ = 0.034
φ and ω scans	θ_max_ = 25.1°, θ_min_ = 2.0°
Absorption correction: multi-scan (*SADABS*; Sheldrick, 1996)	*h* = −7→10
*T*_min_ = 0.960, *T*_max_ = 0.980	*k* = −12→13
13334 measured reflections	*l* = −26→30

### Refinement

**Table:**

Refinement on *F*^2^	Secondary atom site location: difference Fourier map
Least-squares matrix: full	Hydrogen site location: inferred from neighbouring sites
*R*[*F*^2^ > 2σ(*F*^2^)] = 0.060	H-atom parameters constrained
*wR*(*F*^2^) = 0.186	*w* = 1/[σ^2^(*F*_o_^2^) + (0.1097*P*)^2^] where *P* = (*F*_o_^2^ + 2*F*_c_^2^)/3
*S* = 1.05	(Δ/σ)_max_ < 0.001
4452 reflections	Δρ_max_ = 0.33 e Å^−^^3^
290 parameters	Δρ_min_ = −0.36 e Å^−^^3^
0 restraints	Absolute structure: Flack (1983), 1871 Friedel pairs
Primary atom site location: structure-invariant direct methods	Flack parameter: 0.04 (16)

### Special details

**Table:**

Geometry. All e.s.d.'s (except the e.s.d. in the dihedral angle between two l.s. planes) are estimated using the full covariance matrix. The cell e.s.d.'s are taken into account individually in the estimation of e.s.d.'s in distances, angles and torsion angles; correlations between e.s.d.'s in cell parameters are only used when they are defined by crystal symmetry. An approximate (isotropic) treatment of cell e.s.d.'s is used for estimating e.s.d.'s involving l.s. planes.
Refinement. Refinement of *F*^2^ against ALL reflections. The weighted *R*-factor *wR* and goodness of fit *S* are based on *F*^2^, conventional *R*-factors *R* are based on *F*, with *F* set to zero for negative *F*^2^. The threshold expression of *F*^2^ > σ(*F*^2^) is used only for calculating *R*-factors(gt) *etc*. and is not relevant to the choice of reflections for refinement. *R*-factors based on *F*^2^ are statistically about twice as large as those based on *F*, and *R*- factors based on ALL data will be even larger.

### Fractional atomic coordinates and isotropic or equivalent isotropic displacement parameters (Å^2^)

**Table:**

	*x*	*y*	*z*	*U*_iso_*/*U*_eq_	
Cl1	0.8441 (4)	1.17857 (19)	0.06402 (7)	0.1811 (11)	
O1	0.2924 (3)	0.8872 (3)	0.12767 (13)	0.0864 (9)	
H1	0.2020	0.9041	0.1256	0.130*	
O2	1.0028 (3)	0.9464 (3)	0.13070 (14)	0.0927 (10)	
N1	0.7501 (3)	0.9350 (2)	0.13768 (11)	0.0520 (7)	
C1	0.3627 (4)	0.9125 (3)	0.08087 (15)	0.0626 (9)	
C2	0.5208 (3)	0.9060 (3)	0.08034 (14)	0.0536 (9)	
C3	0.5941 (4)	0.9335 (3)	0.03418 (15)	0.0628 (10)	
H3	0.6996	0.9297	0.0333	0.075*	
C4	0.5179 (5)	0.9666 (4)	−0.01089 (15)	0.0746 (12)	
C5	0.3620 (6)	0.9721 (4)	−0.00895 (17)	0.0834 (13)	
H5	0.3081	0.9942	−0.0387	0.100*	
C6	0.2852 (4)	0.9457 (4)	0.03610 (19)	0.0769 (12)	
H6	0.1797	0.9501	0.0366	0.092*	
C7	0.6058 (8)	0.9962 (6)	−0.06040 (18)	0.125 (2)	
H7A	0.6982	0.9512	−0.0613	0.188*	
H7B	0.6297	1.0788	−0.0607	0.188*	
H7C	0.5453	0.9773	−0.0906	0.188*	
C8	0.6007 (3)	0.8750 (3)	0.13155 (14)	0.0512 (8)	
H8	0.5365	0.9094	0.1590	0.061*	
C9	0.6029 (4)	0.7431 (4)	0.14369 (18)	0.0672 (11)	
C10	0.5359 (5)	0.6629 (4)	0.1108 (3)	0.0967 (16)	
H10	0.4909	0.6891	0.0799	0.116*	
C11	0.5336 (7)	0.5430 (5)	0.1227 (4)	0.127 (2)	
H11	0.4850	0.4906	0.1003	0.152*	
C12	0.6012 (8)	0.5029 (6)	0.1664 (4)	0.135 (3)	
H12	0.6032	0.4224	0.1733	0.162*	
C13	0.6684 (7)	0.5812 (6)	0.2016 (3)	0.121 (2)	
H13	0.7133	0.5534	0.2322	0.146*	
C14	0.6677 (5)	0.7034 (5)	0.1903 (2)	0.0923 (14)	
H14	0.7102	0.7568	0.2139	0.111*	
C15	0.8845 (4)	0.8931 (4)	0.12091 (16)	0.0655 (10)	
C16	0.8935 (4)	0.7808 (4)	0.0899 (2)	0.0860 (14)	
H16A	0.9720	0.7875	0.0639	0.129*	
H16B	0.7979	0.7666	0.0729	0.129*	
H16C	0.9162	0.7163	0.1130	0.129*	
C17	0.7476 (4)	1.0531 (3)	0.16311 (13)	0.0566 (8)	
H17	0.8468	1.0892	0.1564	0.068*	
C18	0.6322 (6)	1.1299 (4)	0.13737 (15)	0.0781 (12)	
C19	0.6669 (10)	1.1861 (4)	0.0904 (2)	0.127 (2)	
C20	0.5516 (12)	1.2498 (6)	0.0618 (3)	0.149 (2)	
H20	0.5730	1.2880	0.0304	0.179*	
C21	0.4125 (12)	1.2510 (7)	0.0828 (4)	0.156 (3)	
H21	0.3363	1.2890	0.0640	0.187*	
C22	0.3740 (9)	1.2019 (6)	0.1287 (4)	0.152 (3)	
H22	0.2755	1.2079	0.1416	0.182*	
C23	0.4846 (6)	1.1424 (5)	0.1564 (3)	0.1053 (17)	
H23	0.4599	1.1095	0.1887	0.126*	
C24	0.7295 (5)	1.0460 (4)	0.22294 (17)	0.0842 (12)	
H24	0.6317	1.0095	0.2316	0.101*	
C25	0.8582 (7)	0.9781 (6)	0.24986 (19)	0.1152 (16)	
H25A	0.8233	0.8997	0.2591	0.138*	
H25B	0.9432	0.9701	0.2259	0.138*	
C26	0.9037 (9)	1.0362 (7)	0.2938 (3)	0.150 (2)	
H26A	1.0137	1.0356	0.2958	0.180*	
H26B	0.8646	0.9959	0.3246	0.180*	
C27	0.8506 (8)	1.1554 (7)	0.2936 (2)	0.1364 (19)	
H27A	0.9355	1.2091	0.2893	0.164*	
H27B	0.8008	1.1733	0.3266	0.164*	
C28	0.7391 (7)	1.1699 (5)	0.24837 (19)	0.1022 (14)	
H28A	0.7759	1.2276	0.2233	0.123*	
H28B	0.6404	1.1948	0.2611	0.123*	

### Atomic displacement parameters (Å^2^)

**Table:**

	*U*^11^	*U*^22^	*U*^33^	*U*^12^	*U*^13^	*U*^23^
Cl1	0.303 (3)	0.1376 (15)	0.1031 (11)	−0.0737 (18)	0.0624 (16)	0.0110 (10)
O1	0.0368 (13)	0.119 (3)	0.103 (2)	0.0043 (13)	0.0143 (14)	0.021 (2)
O2	0.0339 (12)	0.114 (2)	0.130 (3)	−0.0049 (14)	0.0073 (15)	−0.034 (2)
N1	0.0335 (12)	0.0595 (17)	0.0629 (16)	0.0023 (12)	−0.0005 (13)	−0.0064 (13)
C1	0.0391 (18)	0.074 (2)	0.075 (2)	−0.0015 (17)	0.0032 (18)	0.0019 (19)
C2	0.0383 (17)	0.056 (2)	0.067 (2)	−0.0048 (14)	0.0028 (15)	−0.0060 (16)
C3	0.053 (2)	0.071 (2)	0.065 (2)	−0.0095 (17)	0.0037 (18)	−0.0108 (18)
C4	0.080 (3)	0.084 (3)	0.060 (2)	−0.031 (2)	0.003 (2)	−0.009 (2)
C5	0.091 (3)	0.087 (3)	0.071 (3)	−0.012 (3)	−0.024 (3)	−0.003 (2)
C6	0.049 (2)	0.088 (3)	0.093 (3)	−0.0030 (19)	−0.020 (2)	−0.005 (2)
C7	0.149 (5)	0.164 (6)	0.063 (3)	−0.065 (4)	0.003 (3)	0.010 (3)
C8	0.0328 (15)	0.057 (2)	0.064 (2)	0.0000 (13)	0.0049 (15)	0.0002 (16)
C9	0.0408 (17)	0.060 (2)	0.101 (3)	−0.0027 (16)	0.0080 (19)	0.008 (2)
C10	0.073 (3)	0.066 (3)	0.151 (5)	−0.008 (2)	0.007 (3)	−0.002 (3)
C11	0.109 (4)	0.060 (3)	0.212 (7)	−0.004 (3)	0.001 (5)	−0.004 (4)
C12	0.109 (5)	0.074 (4)	0.221 (8)	0.019 (4)	0.044 (5)	0.028 (5)
C13	0.103 (4)	0.103 (5)	0.157 (6)	0.023 (4)	0.021 (4)	0.058 (5)
C14	0.075 (3)	0.093 (4)	0.109 (3)	0.009 (2)	0.010 (3)	0.030 (3)
C15	0.0380 (19)	0.079 (3)	0.080 (2)	0.0035 (18)	0.0028 (17)	−0.008 (2)
C16	0.049 (2)	0.093 (3)	0.116 (3)	0.013 (2)	0.022 (2)	−0.024 (3)
C17	0.0516 (18)	0.060 (2)	0.058 (2)	0.0015 (18)	−0.0008 (17)	−0.0073 (16)
C18	0.113 (3)	0.055 (2)	0.066 (2)	0.020 (2)	−0.028 (2)	−0.0157 (19)
C19	0.227 (6)	0.063 (3)	0.091 (3)	0.020 (4)	−0.053 (4)	−0.011 (3)
C20	0.237 (7)	0.098 (4)	0.111 (4)	0.032 (5)	−0.063 (5)	−0.011 (3)
C21	0.215 (7)	0.099 (4)	0.153 (6)	0.060 (5)	−0.088 (6)	−0.031 (4)
C22	0.152 (5)	0.115 (5)	0.187 (6)	0.066 (4)	−0.051 (5)	−0.060 (4)
C23	0.085 (3)	0.088 (3)	0.143 (4)	0.044 (3)	−0.048 (3)	−0.046 (3)
C24	0.082 (3)	0.106 (3)	0.065 (2)	0.003 (2)	−0.019 (2)	−0.016 (2)
C25	0.129 (4)	0.132 (4)	0.084 (3)	0.010 (3)	−0.048 (3)	−0.006 (3)
C26	0.147 (4)	0.177 (5)	0.126 (4)	0.022 (4)	−0.053 (3)	−0.028 (4)
C27	0.133 (4)	0.156 (4)	0.120 (3)	0.000 (4)	−0.044 (3)	−0.042 (3)
C28	0.102 (3)	0.126 (3)	0.078 (3)	0.010 (3)	−0.015 (3)	−0.037 (3)

### Geometric parameters (Å, °)

**Table:**

Cl1—C19	1.701 (9)	C14—H14	0.9300
O1—C1	1.374 (5)	C15—C16	1.501 (6)
O1—H1	0.8200	C16—H16A	0.9600
O2—C15	1.230 (4)	C16—H16B	0.9600
N1—C15	1.345 (4)	C16—H16C	0.9600
N1—C17	1.488 (4)	C17—C18	1.490 (5)
N1—C8	1.489 (4)	C17—C24	1.535 (6)
C1—C6	1.382 (6)	C17—H17	0.9800
C1—C2	1.394 (5)	C18—C19	1.391 (8)
C2—C3	1.377 (5)	C18—C23	1.394 (7)
C2—C8	1.524 (5)	C19—C20	1.443 (9)
C3—C4	1.382 (6)	C20—C21	1.337 (12)
C3—H3	0.9300	C20—H20	0.9300
C4—C5	1.374 (7)	C21—C22	1.340 (11)
C4—C7	1.518 (6)	C21—H21	0.9300
C5—C6	1.366 (6)	C22—C23	1.379 (9)
C5—H5	0.9300	C22—H22	0.9300
C6—H6	0.9300	C23—H23	0.9300
C7—H7A	0.9600	C24—C25	1.532 (7)
C7—H7B	0.9600	C24—C28	1.550 (7)
C7—H7C	0.9600	C24—H24	0.9800
C8—C9	1.528 (5)	C25—C26	1.361 (8)
C8—H8	0.9800	C25—H25A	0.9700
C9—C10	1.370 (7)	C25—H25B	0.9700
C9—C14	1.394 (6)	C26—C27	1.430 (10)
C10—C11	1.393 (8)	C26—H26A	0.9700
C10—H10	0.9300	C26—H26B	0.9700
C11—C12	1.343 (11)	C27—C28	1.523 (8)
C11—H11	0.9300	C27—H27A	0.9700
C12—C13	1.394 (10)	C27—H27B	0.9700
C12—H12	0.9300	C28—H28A	0.9700
C13—C14	1.415 (8)	C28—H28B	0.9700
C13—H13	0.9300		
			
C1—O1—H1	109.5	C15—C16—H16C	109.5
C15—N1—C17	118.0 (3)	H16A—C16—H16C	109.5
C15—N1—C8	125.6 (3)	H16B—C16—H16C	109.5
C17—N1—C8	116.3 (2)	N1—C17—C18	110.2 (3)
O1—C1—C6	123.5 (3)	N1—C17—C24	112.8 (3)
O1—C1—C2	116.6 (3)	C18—C17—C24	113.4 (3)
C6—C1—C2	119.9 (4)	N1—C17—H17	106.7
C3—C2—C1	117.7 (3)	C18—C17—H17	106.7
C3—C2—C8	124.6 (3)	C24—C17—H17	106.7
C1—C2—C8	117.7 (3)	C19—C18—C23	117.2 (5)
C2—C3—C4	122.9 (3)	C19—C18—C17	119.8 (5)
C2—C3—H3	118.5	C23—C18—C17	122.8 (4)
C4—C3—H3	118.5	C18—C19—C20	120.6 (8)
C5—C4—C3	117.9 (4)	C18—C19—Cl1	121.2 (5)
C5—C4—C7	122.0 (4)	C20—C19—Cl1	118.1 (6)
C3—C4—C7	120.2 (4)	C21—C20—C19	116.6 (8)
C6—C5—C4	121.0 (4)	C21—C20—H20	121.7
C6—C5—H5	119.5	C19—C20—H20	121.7
C4—C5—H5	119.5	C20—C21—C22	125.2 (8)
C5—C6—C1	120.6 (4)	C20—C21—H21	117.4
C5—C6—H6	119.7	C22—C21—H21	117.4
C1—C6—H6	119.7	C21—C22—C23	118.2 (8)
C4—C7—H7A	109.5	C21—C22—H22	120.9
C4—C7—H7B	109.5	C23—C22—H22	120.9
H7A—C7—H7B	109.5	C22—C23—C18	122.0 (7)
C4—C7—H7C	109.5	C22—C23—H23	119.0
H7A—C7—H7C	109.5	C18—C23—H23	119.0
H7B—C7—H7C	109.5	C25—C24—C17	113.2 (4)
N1—C8—C2	113.1 (3)	C25—C24—C28	103.2 (4)
N1—C8—C9	114.5 (3)	C17—C24—C28	111.2 (4)
C2—C8—C9	113.9 (3)	C25—C24—H24	109.7
N1—C8—H8	104.6	C17—C24—H24	109.7
C2—C8—H8	104.6	C28—C24—H24	109.7
C9—C8—H8	104.6	C26—C25—C24	110.1 (5)
C10—C9—C14	118.9 (4)	C26—C25—H25A	109.6
C10—C9—C8	121.4 (4)	C24—C25—H25A	109.6
C14—C9—C8	119.6 (4)	C26—C25—H25B	109.6
C9—C10—C11	121.4 (6)	C24—C25—H25B	109.6
C9—C10—H10	119.3	H25A—C25—H25B	108.2
C11—C10—H10	119.3	C25—C26—C27	111.0 (6)
C12—C11—C10	120.3 (7)	C25—C26—H26A	109.4
C12—C11—H11	119.8	C27—C26—H26A	109.4
C10—C11—H11	119.8	C25—C26—H26B	109.4
C11—C12—C13	120.4 (6)	C27—C26—H26B	109.4
C11—C12—H12	119.8	H26A—C26—H26B	108.0
C13—C12—H12	119.8	C26—C27—C28	108.4 (5)
C12—C13—C14	119.5 (6)	C26—C27—H27A	110.0
C12—C13—H13	120.3	C28—C27—H27A	110.0
C14—C13—H13	120.3	C26—C27—H27B	110.0
C9—C14—C13	119.4 (6)	C28—C27—H27B	110.0
C9—C14—H14	120.3	H27A—C27—H27B	108.4
C13—C14—H14	120.3	C27—C28—C24	104.7 (4)
O2—C15—N1	120.4 (3)	C27—C28—H28A	110.8
O2—C15—C16	118.6 (3)	C24—C28—H28A	110.8
N1—C15—C16	120.9 (3)	C27—C28—H28B	110.8
C15—C16—H16A	109.5	C24—C28—H28B	110.8
C15—C16—H16B	109.5	H28A—C28—H28B	108.9
H16A—C16—H16B	109.5		
			
O1—C1—C2—C3	−178.6 (3)	C8—N1—C15—O2	−175.7 (4)
C6—C1—C2—C3	−0.1 (5)	C17—N1—C15—C16	−173.5 (4)
O1—C1—C2—C8	−1.6 (5)	C8—N1—C15—C16	4.4 (6)
C6—C1—C2—C8	176.9 (3)	C15—N1—C17—C18	127.5 (4)
C1—C2—C3—C4	−0.1 (6)	C8—N1—C17—C18	−50.6 (4)
C8—C2—C3—C4	−176.9 (3)	C15—N1—C17—C24	−104.7 (4)
C2—C3—C4—C5	0.1 (6)	C8—N1—C17—C24	77.2 (4)
C2—C3—C4—C7	179.8 (4)	N1—C17—C18—C19	−80.3 (5)
C3—C4—C5—C6	0.0 (7)	C24—C17—C18—C19	152.2 (4)
C7—C4—C5—C6	−179.7 (5)	N1—C17—C18—C23	95.1 (4)
C4—C5—C6—C1	−0.1 (7)	C24—C17—C18—C23	−32.4 (5)
O1—C1—C6—C5	178.6 (4)	C23—C18—C19—C20	−2.8 (7)
C2—C1—C6—C5	0.2 (6)	C17—C18—C19—C20	172.9 (5)
C15—N1—C8—C2	−89.0 (4)	C23—C18—C19—Cl1	179.0 (4)
C17—N1—C8—C2	88.9 (3)	C17—C18—C19—Cl1	−5.3 (6)
C15—N1—C8—C9	43.7 (5)	C18—C19—C20—C21	−0.2 (9)
C17—N1—C8—C9	−138.3 (3)	Cl1—C19—C20—C21	178.1 (6)
C3—C2—C8—N1	30.9 (5)	C19—C20—C21—C22	2.6 (12)
C1—C2—C8—N1	−145.9 (3)	C20—C21—C22—C23	−1.8 (12)
C3—C2—C8—C9	−102.2 (4)	C21—C22—C23—C18	−1.5 (9)
C1—C2—C8—C9	81.0 (4)	C19—C18—C23—C22	3.7 (7)
N1—C8—C9—C10	−133.2 (4)	C17—C18—C23—C22	−171.8 (5)
C2—C8—C9—C10	−0.9 (5)	N1—C17—C24—C25	60.2 (5)
N1—C8—C9—C14	49.9 (5)	C18—C17—C24—C25	−173.7 (4)
C2—C8—C9—C14	−177.7 (3)	N1—C17—C24—C28	175.9 (3)
C14—C9—C10—C11	−1.1 (7)	C18—C17—C24—C28	−58.1 (5)
C8—C9—C10—C11	−177.9 (4)	C17—C24—C25—C26	136.6 (6)
C9—C10—C11—C12	−1.7 (9)	C28—C24—C25—C26	16.2 (7)
C10—C11—C12—C13	2.9 (10)	C24—C25—C26—C27	−16.4 (10)
C11—C12—C13—C14	−1.4 (10)	C25—C26—C27—C28	9.3 (10)
C10—C9—C14—C13	2.6 (7)	C26—C27—C28—C24	1.3 (8)
C8—C9—C14—C13	179.5 (4)	C25—C24—C28—C27	−9.9 (6)
C12—C13—C14—C9	−1.4 (8)	C17—C24—C28—C27	−131.6 (5)
C17—N1—C15—O2	6.4 (5)		

### Hydrogen-bond geometry (Å, °)

**Table:**

*D*—H···*A*	*D*—H	H···*A*	*D*···*A*	*D*—H···*A*
O1—H1···O2^i^	0.82	1.82	2.637 (3)	172

Symmetry codes: (i) *x*−1, *y*, *z*.

## References

[bb1] Ali, M. A., Mirza, A. H., Butcher, R. J. & Tarafder, M. T. H. (2002). *Inorg. Biochem.* **92**, 141–148. 10.1016/s0162-0134(02)00559-7 12433421

[bb2] Bruker (1999). *SMART* and *SAINT* Bruker AXS Inc., Madison, Wisconsin, USA.

[bb3] Calligaris, M., Nardin, G. & Randaccio, L. (1972). *Coord. Chem. Rev.* **7**, 385–403.

[bb4] Cukurovali, A., Yilmaz, I., Ozmen, H. & Ahmedzade, M. (2002). *Transition Met. Chem.* **27**, 171–176.

[bb5] Flack, H. D. (1983). *Acta Cryst.* A**39**, 876–881.

[bb6] Kargar, H., Jamshidvand, A., Fun, H.-K. & Kia, R. (2009). *Acta Cryst.* E**65**, m403–m404. 10.1107/S1600536809008721 PMC2968910 21582349

[bb7] Sheldrick, G. M. (1996). *SADABS* University of Gottingen, Germany.

[bb8] Sheldrick, G. M. (2008). *Acta Cryst.* A**64**, 112–122. 10.1107/S0108767307043930 18156677

[bb9] Sriram, D., Yogeeswari, P., Myneedu, N. S. & Saraswat, V. (2006). *Bioorg. Med. Chem. Lett.* **16**, 2127–2129. 10.1016/j.bmcl.2006.01.050 16458506

[bb10] Takenaka, N., Huang, Y. & Rawal, V. H. (2002). *Tetrahedron*, **58**, 8299–8305.

[bb11] Varlamov, A. V., Zubkov, F. I., Boltukhina, E. V., Sidorenko, N. V. & Borisov, R. S. (2003). *Tetrahedron Lett.* **44**, 3641–3643.

[bb12] Yang, X.-F., Zhang, G.-Y., Zhang, Y., Zhao, J.-Y. & Wang, X.-B. (2005). *Acta Cryst.* C**61**, o262–o264. 10.1107/S0108270105005676 15805646

[bb13] Zhang, G.-Y., Liao, Y.-Q., Wang, Z.-H., Nohira, H. & Hirose, T. (2003). *Tetrahedron Asymmetry*, **14**, 3297–3300.

